# Using Animated Videos to Promote the Accessibility and Understandability of Package Leaflets: Retrospective Observational Study Evaluating the First Year of Implementation

**DOI:** 10.2196/40914

**Published:** 2023-05-04

**Authors:** Liselot N van den Berg, Niels H Chavannes, Jiska J Aardoom

**Affiliations:** 1 Department of Public Health and Primary Care Leiden University Medical Center Leiden Netherlands; 2 National eHealth Living Lab Leiden Netherlands

**Keywords:** package leaflet, health literacy, implementation, online library, animated videos, accessibility, understandability, comprehension, eHealth, medication, health communication

## Abstract

**Background:**

The medication package leaflet is the most used and trusted source of information in the home situation but is often incomprehensible for individuals, especially for those with limited health literacy. The platform “Watchyourmeds” comprises a web-based library with over 10,000 animated videos that explain the most essential information from the package leaflet in an unambiguous and simple manner to increase the accessibility and understandability of package leaflets.

**Objective:**

This study aimed to investigate Watchyourmeds in the Netherlands from a user perspective during the first year of implementation by investigating (1) usage data, (2) self-reported user experiences, and (3) the preliminary and potential impact on medication knowledge.

**Methods:**

This was a retrospective observational study. The first aim was investigated by examining objective user data from 1815 pharmacies from the first year of implementation of Watchyourmeds. User experiences (second aim) were investigated by examining individuals’ completed self-report questionnaires (n=4926) that they received after completing a video. The preliminary and potential impact on medication knowledge (third aim) was investigated by examining users’ self-report questionnaire data (n=67) that assessed their medication knowledge about their prescribed medication.

**Results:**

Nearly 1.8 million videos have been distributed to users by over 1400 pharmacies, with monthly numbers increasing to 280,000 in the last month of the implementation year. Most users (4444/4805, 92.5%) indicated to have fully understood the information presented in the videos. Female users reported more often to have fully understood the information than male users (*χ*^2^_4_=11.5, *P*=.02). Most users (3662/4805, 76.2%) said that they did not think any information was missing in the video. Users with a lower educational level stated more often (1104/1290, 85.6%) than those with a middle (984/1230, 80%) or higher (964/1229, 78.4%) educational level that they did not seem to be missing any information in the videos (*χ*^2^_12_=70.6, *P*<.001). A total of 84% (4142/4926) of the users stated that they would like to use Watchyourmeds more often and for all their medication, or would like to use it most of the time. Male users and older users stated more often that they would use Watchyourmeds again for other medication than the female (*χ*^2^_3_=25.0, *P*<.001) and younger users (*χ*^2^_3_=38.1, *P*<.001), respectively. Almost 88% (4318/4926) of the users would recommend the web-based library to friends, family, or acquaintances. Regarding the third aim, results showed that 73.8% (293/397) of the questions assessing users’ medication knowledge were answered correctly.

**Conclusions:**

The results of this study suggest that a web-based library with animated videos is a valuable and acceptable addition to stand-alone package leaflets to increase the understanding and accessibility of medication information.

## Introduction

Patients do not always pick up their medicines from the pharmacy or take their prescription as prescribed. Patients collect around 48% to 66% of every 100 prescriptions at the pharmacy, and only 15% to 20% are refilled as prescribed [1]. Furthermore, it is estimated that approximately 25% of patients are not taking their medicines as prescribed [2]. This percentage is even higher in patients with chronic illness, at approximately 50% [3]. According to the World Health Organization, nonadherence is “a worldwide problem of striking magnitude” [4]. Nonadherence results in unnecessary burdening of patients (eg, further disease progression or delayed recovery [5] and a higher risk of hospitalization [5,6]), wastage of medicines, and a substantial economic burden for society [7]. Regarding the latter, the overall cost of nonadherence is estimated around US $290 billion annually [1], of which US $100 billion are related to hospital admissions [8].

Health professionals and pharmacists have an essential role in informing patients about medication and the package leaflets [9]. Indeed, a lack of shared decision-making and a lack of trust in the health care professional are related to medication nonadherence [10]. Proper use of medicines furthermore requires sufficient knowledge and understanding of the medicine. One aspect associated with poor medication adherence is low health literacy [5,8,11]. Health literacy can be defined as “the degree to which individuals have the capacity to obtain, process, and understand basic health information and services needed to make appropriate health decisions” [12]. Indeed, most individuals with limited health literacy do not understand their prescribed medication, lacking knowledge about why they have to take it, when and how to use it, and its associated risks [11,13]. According to different health literacy studies in Europe, up to 50% of the population has limited health literacy [14,15]. The package leaflet is the most used and trusted source of information in the home situation but is often incomprehensible for individuals with limited health literacy skills [15,16]. The results of an Italian study in pharmacies showed that most individuals (84%) read the package leaflet but more than half of them (54%) reported the leaflet not being easy to understand [17].

As package leaflets play an essential role in safe and effective medication usage, a European readability guideline was developed in 2009 [18]. This guideline requires that 80% of the people should be able to answer each question about the label or package leaflet correctly. These questions reflect general issues (eg, what to do when you miss a dose?), specific issues (eg, what specific side effects may occur?), and essential safety issues. Unfortunately, a study published in 2016 that evaluated 36 package leaflets showed that none of these leaflets succeeded in meeting the 80% criteria of the European guideline [19]. Moreover, the authors compared the readability of the leaflets before and after the implementation of the European guideline. They concluded that there were no improvements in terms of the readability of the package leaflets. The literature identifies various usability and readability problems, such as the leaflet being too long, the text structure being unclear, and critical information being buried in the amount of text [20]. Hence, leaflets are often only accessible to a limited number of higher educated people and do not promote health literacy, which may deepen inequalities in health [21].

A way to improve comprehension and medication adherence is by adjusting the information in the package leaflets. Especially for people with limited health literacy skills, using pictograms—picture-based instructions instead of text-based instructions—could help increase their understanding of the information about the medication. Indeed, several studies show that the combination of pictograms and text is more effective than using either one alone [17,22-24]. In line with this, aiming to increase the accessibility of package leaflets for individuals with limited health literacy skills, the Dutch company Careanimations developed a platform called “Watchyourmeds,” comprising a web-based library with over 10,000 animated videos for the Netherlands alone [25]. These videos explain the essential information from the medication package leaflet in an unambiguous, simple, understandable, and accessible manner. Just as pictograms, videos are considered suitable for transferring complex information, such as in package leaflets, because written text can be replaced by simple spoken language and the information can be supported visually [26,27]. In 2019, Watchyourmeds was implemented at the national level in Dutch pharmacies. At that time, the web-based library was available for more than 95% of all newly prescribed medicines in primary care and approximately 80% of all available medicines in the Netherlands. During January 2019, around 80% of the community pharmacies and 70% of the outpatient pharmacies used Watchyourmeds. Its implementation was evaluated in 153 pharmacies, demonstrating Watchyourmeds to be a valuable addition to the currently available sources of medication information, especially at individuals’ first dispensing [28].

This study aimed to supplement the evaluation of the Dutch pharmacy perspective by investigating Watchyourmeds from a user perspective during the first year of implementation. More specifically, this study aimed to evaluate the first year of implementation by investigating (1) user data for the year 2019 (ie, the year of implementation of Watchyourmeds), (2) self-reported user experiences (ie, accessibility and understandability) during that year (of only users who completed a Watchyourmeds video), and (3) the preliminary and potential impact on medication knowledge (ie, understandability). This study provides a relevant opportunity to advance the understanding of an innovative tool that could improve the accessibility and understandability of the package leaflet. Exploring the implementation could benefit both users and health care professionals, such as pharmacists and physicians.

## Methods

### Study Design

This was a retrospective observational study because it analyzed already existing data since a past event (ie, the implementation of Watchyourmeds). The first study aim was investigated by examining objective user data of individuals from the year of implementation of Watchyourmeds (ie, 2019). User evaluations (second aim) were investigated by examining individuals’ completed self-report questionnaires regarding their experiences with Watchyourmeds. The preliminary and potential effect on medication knowledge (third aim) was investigated by examining users’ self-report questionnaire data regarding medication knowledge about their medicines.

### Ethical Considerations

This study did not fall within the scope of the Dutch Medical Research Involving Human Subjects Act by the medical ethics committee of Leiden University Medical Centre (G20.034). Subsequently, a declaration of no objection was obtained from the medical ethics committee. The website of Watchyourmeds includes a privacy statement stating that user data may be examined for statistical and scientific purposes. Furthermore, users are informed that all data are rendered anonymous before they are examined, meaning that the respective researchers could not trace any data back to specific individuals. The statement also reports that the above applies to information one may enter when voluntarily completing questionnaires on the website.

### Web-Based Platform “Watchyourmeds”

The platform Watchyourmeds comprises a web-based library with over 10,000 animated videos for the Netherlands alone. The content of the first videos was developed by pharmacists and physicians, together with pharmacy consultancies. Language ambassadors from Pharos, a Dutch institute with expertise in health disparities, tested these videos iteratively. Afterward, the content was piloted with a group of pharmacists. A standard operating procedure was developed by the developers of Watchyourmeds accordingly. The current procedure is that 2 pharmacy master students get the first extraction from the package leaflet, and a pharmacist reviews this. Then a senior pharmacist authorizes the written text and checks whether the template (included in the standard operating procedure, which is tested by individuals with low literacy levels) is adequately used and whether the language is comprehensible. The videos are entirely based on the package leaflet’s content (ie, have the same setup as the package leaflet) and contain the most essential information (eg, why and how the medication is used and related side effects) from the leaflet in an understandable spoken language. This way users know what to do, what to expect, and which aspects need special attention. Hence, the animated videos add an explanation to the official leaflet but do not replace these.

In each video, approximately 800 words are used. Watching an entire video generally takes around 5 minutes and 30 seconds. The videos display a consulting room with a pharmacist and patient conducting a dialogue (see Multimedia Appendix 1). During the presented consult, the patient asks questions and the pharmacist answers these accordingly. The scripts used in the videos consist of both general information, such as the advice to report allergies to a doctor, and medicine-specific information. The information includes the following topics: (1) What is this medicine for?, (2) When should I not use it?, (3) When should I be careful?, (4) Other medicines, (5) Food and drinks, (6) Pregnancy and breastfeeding, (7) Driving, (8) How to use it?, (9) Used too much?, (10) Forgotten to use it or stopping?, (11) Side effects, and (12) How to store it? An interactive menu is available while watching the video (see Multimedia Appendix 1), enabling the user to choose and alternate between the different topics easily. All videos are personalized for the indication of the prescription (eg, oral and inhalation), gender, and age to allow users to associate themselves with the setting. For example, information about medication use during pregnancy and breastfeeding is never shown to male users. Videos are available in 4 languages: Dutch, English, Arabic, and Turkish.

### Procedure and Participant Flow

In the Netherlands, pharmacists stock a wide range of prescription and over-the-counter medication. Prescription medicines are medicines that individuals can only get if they have a doctor’s prescription, for example, from one’s general practitioner, dentist, or midwifery professional. Over-the-counter medicines can be sold to individuals directly without a prescription. When individuals collected their prescribed medication or over-the-counter medicines at the Dutch pharmacy, they were offered Watchyourmeds via one of the following three distribution methods: (1) a flyer, (2) a medication-specific link that was distributed through a patient portal or personal health record, or (3) a medication-specific link that was distributed through email or mobile text messaging. The type of distribution method depended on the pharmacy resources being available at the time of the research. When individuals received a flyer, they were instructed how to find their specific prescribed medication on the platform of Watchyourmeds through a code, the name of the medication, or a barcode scan [28]. For example, they could find a QR code on the flyer, which they could scan, or go immediately to the Watchyourmeds webpage and scan the code on their medicine box or search by the name of the medicine on the landing page.

Individuals who accessed the platform could choose from among 4 different languages: Dutch, English, Arabic, and Turkish. Afterward, depending on the link they received from the pharmacy, they selected their gender, age group, and the reason or indication for which they were prescribed the medicine. These selections ensured that they were offered the correct video matching their personal situation. After watching the video, users were offered the option to create an account to save their video. Through a privacy statement, users were informed that some of their data (eg, how they were using the website and on what device) in the form of cookies would be used for statistical and scientific purposes that could subsequently be used to help assess and improve the user experience. Individuals were also informed that their data would be rendered entirely anonymous before being examined and hence could not be traced back to specific individuals. After watching a video, individuals could also voluntarily complete an evaluation questionnaire or knowledge test (see section “Questionnaires and Data” for more details).

### Questionnaires and Data

All data were collected by the company Careanimations on a secured server. For this study, Careanimations shared the anonymized data (ie, having removed personal identifiers such as email addresses and making sure that the data could not be traced back to a specific individual’s data) with the *Watchyourmeds* foundation, responsible for distributing Watchyourmeds in the Netherlands, which subsequently shared these data with the involved researchers of the Leiden University Medical Center.

#### First Aim—Objective User Data

Objective user data were extracted from a pharmacy-level data set including 1815 Dutch pharmacies. These data were collected via the underlying software; individuals received a Watchyourmeds link from their pharmacist, which is connected to an individual pharmacist (ie, the pharmacist code is locked into this link). When individuals did not receive a link to Watchyourmeds, they still needed to choose their pharmacy after going to the webpage. Data were included from both nonregistered and registered users, including data about how many animated videos were watched during the year 2019, as well as general sociodemographic data: gender, age category (younger or older than 55 years), and language (Dutch, English, Arabic, or Turkish). Starting from October 2019, it became possible to collect data about the usage of the interactive menus in videos. These data were collected until the end of the year and included 766,984 viewed videos. Because of an unknown technical difficulty, 30,000 viewed videos regarding the interactive menu were lost.

#### Second Aim—User Experiences

As specified in the participant flow, registered users were asked to complete an evaluation questionnaire after watching a video by clicking on a button. The evaluation questionnaire included 8 multiple-choice questions. One question assessed one’s educational level (note: only for Dutch users). The other 7 questions assessed a variety of aspects related to individuals’ satisfaction with Watchyourmeds, for example, asking whether one would recommend the platform to family and friends, whether one perceived Watchyourmeds as a valuable supplement to the information that one would normally receive from their health care professional or pharmacist, and the degree to which one has understood the information that was presented in the video. An example question is “After watching Watchyourmeds, do you think you have enough knowledge to use this medicine properly?” with answer categories (1) I certainly know enough, (2) I probably know enough, (3) I still have too little knowledge, and (4) I do not know if I have enough knowledge.

A data set was used to investigate user experiences (ie, accessibility and understandability), including data from users who voluntarily completed the evaluation questionnaire. The data set included data from 4926 Dutch users. Not all users completed the questionnaire. In addition, 9 users filled out the questionnaire in the English language (8 people completed it), 3 in the Turkish language, and 1 person in the Arabic language. The subgroup analyses based on the educational level were conducted on a subsample of 4324 users; from the 4926 Dutch users, for only 4335 users the educational level was known, and 21 duplicates were removed.

#### Third Aim—Preliminary and Potential Impact on Medication Knowledge

As specified in the participant flow, users were enabled to voluntarily complete an evaluation questionnaire and a knowledge test after watching a video by clicking on a button. The knowledge tests comprised 5 to 8 (depending on the specific medicine) multiple-choice questions related to how to use the medicine correctly and its side effects. For example, “What do you do if you have taken too much of this medicine?” and “What do you do if you forget to take this medicine?” In 2019, the knowledge tests were only available for 10 commonly prescribed medicines: acetylsalicylic acid, citalopram, enalapril, gliclazide, levothyroxine, metformin, metoprolol, omeprazole, salbutamol, and simvastatin.

A data set comprising data of completed knowledge tests was used to investigate the preliminary and potential impact on medication knowledge (ie, understandability). This data set originally included 71 users who completed 1 or more knowledge tests. However, 4 users were excluded because they filled in the knowledge tests about the same medicine more than once; therefore, we considered these results unreliable. This resulted in a final data set with 67 users who answered 397 questions about their prescribed medication after watching a video.

### Statistical Analysis

For all 3 study aims, descriptive analyses were conducted, more specifically, means and SDs and counts and percentages where appropriate. Regarding the second and third aim, chi-square tests were conducted to exploratively examine potential differences between subgroups based on gender, age category, and educational level. The data were analyzed using the statistical software SPSS (version 25.0; IBM Corp) [29].

## Results

### First Aim—Objective User Data

During 2019, a total of 1,788,890 videos were watched by users from 1474 of the 1815 pharmacies (81%) in the Netherlands. The other pharmacies (341/1815, 19%) had a Watchyourmeds subscription but did not distribute any flyers or links to the platform. In January 2019, a total of 13,683 videos were watched by users from 105 pharmacies, and these numbers increased during the year. In December 2019, a total of 280,401 videos were watched by users from 1268 pharmacies. More information about the number of pharmacies participating, the number of distributed videos, and distribution methods can be found in Figures S1, S2, and S3, respectively, of Multimedia Appendix 2. Around half of the users were female (1,002,368/1,788,890, 56%). Two-thirds (1,139,730/1,788,890, 63.7%) of the users were 55 years or older. Table 1 shows an overview of the demographic characteristics of the different data sets used in this study.

Concerning the language of accessed videos, almost all videos were viewed in Dutch (1,784,058/1,788,890, 99.7%). Only a small minority of all videos were watched in English (n=2640), Arabic (n=1237), and Turkish (n=955). Users from 360 different pharmacies watched videos in another language than Dutch. In these pharmacies, on average, 7 videos were watched in English, 3 in Turkish, and 3 in Arabic. Three different distribution methods were used in 2019. The most commonly used method to offer Watchyourmeds was through a flyer (641/1577, 40.6%).

When assessing the usage data for the interactive menu, data showed that most users (611,302/766,984, 79.7%) did not use the interactive menu. Of the users who did use the interactive menu (155,682/766,984, 20.3%), the most frequently selected topics were “Side effects” (21,438/155,682, 13.8%), “What is this medicine for?” (17,658/155,682, 11.3%), and “How to use it?” (17,441/155,682, 11.2%). Other topics ranged between 7.4% and 10.9% except for “Pregnancy and breastfeeding” (2357/155,682, 1.5%) and “How to store it?” (937/155,682, 0.6%). Both these latter topics are disabled when they do not apply to a specific target group or medicine.

**Table 1:** Sociodemographic characteristics of users in the different data sets.

Characteristic		Subsample objective user data	Subsample evaluation questionnaire^a^	Subsample knowledge tests
**Gender, n (%)**				
	Male	786,522 (44)	21,790 (49.7)	34 (50.7)
	Female	1,002,368 (56)	21,988 (50.2)	33 (49.3)
	Unknown	—^b^	54 (0.1)	—
**Age group, n (%)**				
	Young (≤54 years)	649,160 (36.3)	8754 (20)	22 (32.8)
	Old (≥55 years)	1,139,730 (63.7)	31,550 (72)	45 (67.2)
	Unknown	—	3528 (8)	—
**Language, n (%)**				
	Dutch	1,784,058 (99.7)	4926 (99.7)	67 (100)^c^
	English	2640 (0.1)	9 (0.1)	—
	Arabic	1237 (0.1)	3 (0.1)	—
	Turkish	955 (0.1)	1 (0.1)	—
**Education level, n (%)**				
	No education or elementary school	—	92 (2.1)	—
	Lower education	—	1355 (31.1)	—
	Middle education	—	1300 (30.1)	—
	Higher education	—	1314 (30.4)	—
	Other^d^	—	145 (3.4)	—

^a^The subsample evaluation questionnaire had a long format. Each row existed out of 1 of the 8 multiple-choice questions and stated the gender and age category of the user.

^b^Not applicable.

^c^The knowledge tests were only available in the Dutch language.

^d^Unknown.

### Second Aim—User Experiences

Of the users who completed the evaluation questionnaire, approximately half were female and three-quarters of the users were 55 years or older (see Table 1). The different educational levels (ie, low, middle, and high) were evenly distributed, around one-third in each group, respectively, except for the groups comprising people with no or elementary school education and people in the group “other.” The category “other” was excluded in the analyses because of an unknown educational level. Furthermore, only the results regarding the evaluation questionnaire on user experiences in the Dutch language are presented in this paper, given the limited data available in the other languages (see Table 1). The results regarding the evaluation questions can be found in Table 2. The results of the subgroup analyses based on gender, age, and educational level can be found in Tables S1, S2, and S3, respectively, of Multimedia Appendix 3.

**Table 2:** Questionnaire results regarding the evaluation of the web-based platform Watchyourmeds.

Questions and corresponding answer options		Values, n (%)
**To what extent did you understand the information in this Watchyourmeds?**		
	I did not understand any of it	41 (0.9)
	I understood very little	12 (0.2)
	I understood about half of it	16 (0.3)
	I understood most of the information	292 (6.1)
	I understood the information fully	4444 (92.5)
**Did Watchyourmeds add to the information about your medication that you received from your health care professional?**		
	No	1370 (28.5)
	Yes	3435 (71.5)
**Did you think there was information missing from this Watchyourmeds?**		
	I did not think anything was missing	3662 (76.2)
	I felt very little was missing	634 (13.2)
	I thought a number of things were missing	175 (3.6)
	I missed a lot	46 (1)
**Are you going to take or use the medication for which you looked at this Watchyourmeds?**		
	I do not know	193 (4)
	No	80 (1.7)
	Yes	4532 (94.3)
**After watching Watchyourmeds, do you think you have enough knowledge to use this medicine properly?**		
	I certainly know enough	3749 (76.1)
	I probably know enough	905 (18.4)
	I still have too little knowledge	72 (1.5)
**Do you think you will use Watchyourmeds again, and also for other medication?**		
	No, I will never use Watchyourmeds again	155 (3.1)
	I do not think I will use Watchyourmeds very often	629 (12.8)
	Yes, I think I will usually use Watchyourmeds	1785 (36.2)
	Yes, I will use Watchyourmeds for all my medication	2357 (47.8)
**Are you likely to recommend Watchyourmeds to friends, family, or acquaintances?**		
	0 (No, definitely not)	149 (3)
	1	37 (0.8)
	2	31 (0.6)
	3	62 (1.3)
	4	56 (1.1)
	5	273 (5.5)
	6	236 (4.8)
	7	656 (13.3)
	8	1184 (24)
	9	496 (10.1)
	10 (Yes, definitely)	1746 (35.6)

In general, the majority of users (4444/4805, 92.5%) indicated to have fully understood the information presented in the video (see Table 2). The results of the subgroup analyses (see Multimedia Appendix 3) showed that a relatively higher percentage of female users reported to have fully understood the information as presented in the videos (*χ*^2^_4_=11.5, *P*=.02). There was no significant difference between the 2 age categories in terms of the reported degree of understanding the information in the video (*χ*^2^_4_=2.5, *P*=.65). The proportion of users with no or elementary school education who reported fully understanding the information was relatively lower (73/92, 79.3%) than the proportion as reported in all other subgroups based on educational levels (range 92.5%-94%; *χ*^2^_16_=93.7, *P*<.001).

The majority of the users (3435/4805, 71.5%) indicated Watchyourmeds to be a valuable addition to the medication information they received from their health care professional (see Table 2). The proportion of male users who perceived Watchyourmeds as a valuable addition was relatively higher than that of female users (*χ*^2^_1_=8.0, *P*=.005). Furthermore, the younger age group (≤54 years) more often indicated that Watchyourmeds added to the medication information normally received as compared with the older age group (≥55 years; *χ*^2^_1_=13.6, *P*<.001). All subgroups based on educational levels significantly differed from each other with respect to this item. More specifically, the relative number of individuals who consider Watchyourmeds an addition to the information normally received from their health care professional increases when going from higher to lower educational levels (*χ*^2^_4_=122.7, *P*<.001; for more details, see Multimedia Appendix 3).

Most users (3662/4805, 76.2%) stated that they did not think any information was missing in the video (see Table 2). The results of the subgroup analyses (see Multimedia Appendix 3) showed that several gender differences were found regarding whether one thought that there was information missing in the videos, however, without any clear direction (*χ*^2^_3_=15.6, *P*=.001). Individuals in the older age group reported relatively more often to have not missed any information (*χ*^2^_3_=41.8, *P*<.001). Users with a lower educational level stated more often (1104/1290, 85.6%) than users with a middle (984/1230, 80%) or higher (964/1229, 78.4%) educational level that they did not miss any information (*χ*^2^_12_=70.6, *P*<.001).

Regarding whether individuals would take or use the respective medication in the viewed video, most of the sample answered “yes” (4532/4805, 94.3%; see Table 2). No significant differences were found between males and females (*χ*^2^_2_=2.6, *P*=.27). The proportion of younger users who reported going to take or use the medication after watching the Watchyourmeds video was relatively lower than the proportion of older users who answered to do so (*χ*^2^_2_=6.6, *P*=.04; see Multimedia Appendix 3). Moreover, the proportion of users with a higher educational level who stated going to take or use the medication (1221/1314, 92.9%) was relatively lower than the proportion of users with a lower (1301/1355, 96%) or middle educational level (1236/1300, 95.1%; *χ*^2^_8_=28.1, *P*<.001).

Most users indicated to certainly have sufficient knowledge to correctly use their medication after watching a video (3749/4926, 76.1%; see Table 2). The subgroup analyses (see Multimedia Appendix 3) revealed no significant differences between males and females (*χ*^2^_2_=1.7, *P*=.42). However, younger users relatively less often indicated to certainly know enough to use the medication correctly after watching a video as compared with older users (*χ*^2^_2_=15.2, *P*=.001). The subgroup with a higher educational level more often indicated to still have too little knowledge after watching a video compared with the subgroup with a lower educational level (*χ*^2^_8_=27.6, *P*=.001).

Around half of the users stated that they would use Watchyourmeds more often and for all their medication and approximately one-third considered using it most of the time (see Table 2). Male users stated relatively more often that they would use Watchyourmeds again for other medication than female users (*χ*^2^_3_=25.0, *P*<.001). Similarly, the older users stated this more often than the younger users (*χ*^2^_3_=38.1, *P*<.001). All subgroups based on educational level significantly differed from each other (*χ*^2^_12_=180.3, *P*<.001; see Multimedia Appendix 3).

Most users would likely recommend Watchyourmeds to friends, family, or acquaintances (see Table 2). More specifically, on a scale of 0 (no, definitely not) to 10 (yes, definitely), most users scored between 7 and 10 (4082/4926, 83%).

### Third Aim—Preliminary and Potential Impact on Medication Knowledge

Approximately half of all users who completed the knowledge tests were male, and two-thirds were 55 years or older (see Table 1). Of all the 397 answered questions, 73.8% were answered correctly (n=293). The results of the subgroup analyses revealed no significant differences between males and females (*χ*^2^_1_=0.005, *P*=.95). Furthermore, no significant differences were found between the 2 age groups (*χ*^2^_1_=0.3, *P*=.61).

## Discussion

### Principal Results

This study aimed to provide insight into using animated videos to promote the accessibility and understandability of medication package leaflets during their first year of implementation in the Netherlands. Furthermore, preliminary user perspectives and potential impact on users’ medication knowledge were investigated. The results showed that through over 1400 Dutch pharmacies, nearly 1.8 million videos were watched, with monthly numbers increasing to 280,000 in the last month of the year. Users generally evaluated Watchyourmeds positively among others in terms of understanding the presented information in the videos, as well as it being a valuable addition to the medication information as received from health care professionals. Furthermore, almost 88% of the users would recommend the online library to friends, family, or acquaintances. Finally, results indicated that almost three-quarters of the questions assessing medication knowledge were answered correctly. The results of this study suggest Watchyourmeds to be a valuable addition to stand-alone package leaflets in terms of the accessibility and understandability of medication information.

Regarding preliminary user perspectives, most users stated to have (fully) understood the information as presented in the videos and to not have missed any information: approximately 99% and 76%, respectively. These rates are much more positive than an Italian study assessing the comprehensibility rate of stand-alone package leaflets, showing that 54% of individuals reported the leaflet not being easy to understand [17]. Hence, animated videos seem to improve the accessibility and understandability of medication information. The results of this study seem consistent with previous research demonstrating that animated videos are the best way to communicate complex health information to users, especially those with low health literacy [26,27].

The results suggest Watchyourmeds to be an even more valuable tool to improve the understandability of the package leaflet for individuals with lower levels of education. Specifically, this was reflected in the relatively higher rates of participants indicating Watchyourmeds to add to the information about their medication normally received from their health care professional; those with no or elementary education answered “yes” more often (82/92, 89%) than those with lower (1066/1355, 79%), middle (944/1300, 73%), and higher (798/1314, 61%) educational levels. The overall positive user perspectives of Watchyourmeds are in line with the findings of Meppelink et al [26] that people with limited health literacy had more positive attitudes toward spoken messages.

Despite some subgroup differences based on gender, age, and education with respect to some user perspectives of Watchyourmeds, an important finding is that Watchyourmeds appears to be a positive addition in understanding the package leaflet for a wide range of people. Because leaflets are often incomprehensible for individuals with limited health literacy [13,15,16], there is a possibility of increasing health inequalities [21]. Watchyourmeds may reduce these inequalities by providing easy-to-understand animated information.

### Limitations

The current results should be interpreted in light of several study limitations. One limitation is that, regarding user perspectives, only self-reported data were collected from users who watched a video and afterward voluntarily clicked on a button to complete a questionnaire about user perspectives. There is a chance that the data were collected from a mainly enthusiastic group of users, whereas data from individuals who did not watch a complete video or chose not to fill in the questionnaire are underrepresented. This potentially limits the generalizability of the study findings. Next, social desirability bias may have occurred during the data collection. However, as the data were collected anonymously, this may have reduced the presence of social desirability.

A second limitation is that the knowledge questions were only asked after watching an animated video. Consequently, knowledge levels could not be compared before and after watching a video; therefore, we could not investigate whether knowledge levels were increased. Also, the number of participants who filled in the knowledge tests during 2019 was small (n=67). Hence, the results regarding the third aim (ie, the preliminary and potential impact on medication knowledge) should be carefully interpreted because of limited power.

A third limitation of this study is that data on users’ age were only available as a categorical variable: relatively young users (≤54 years old) and relatively old users (≥55 years old). This categorization is arbitrary, and comparing only 2 age groups may have decreased the chances of identifying any potential nuances in the data.

### Implications for Future Research and Practice

More research is needed to gain a deeper understanding of user perspectives. Qualitative research, such as focus groups or interviews, could be conducted to examine more in-depth experiences with, and needs and improvement possibilities of, Watchyourmeds and to investigate potential similarities and differences between different subgroups based on, for example, educational levels, age, and gender. Additionally, quantitative research with validated questionnaires and the collection of user data (ie, objective data) would be valuable to expand the understanding of user perspectives further. Next, examining the relationship between the usage, user experience, and efficacy of Watchyourmeds on medication knowledge would be interesting.

Moreover, the videos were primarily viewed in Dutch during the implementation year, as the most common distribution methods (ie, flyers and patient portals) were only available or presented in Dutch at the time. Follow-up studies could investigate usage patterns and user perspectives for languages other than Dutch. For example, to investigate which distribution route is mainly used and is considered most feasible for non–Dutch speaking individuals. Since 2019, Watchyourmeds has been containing knowledge tests for almost all medicines, and knowledge tests are offered before and after watching the video to make pre-post testing available. This makes it possible to examine whether knowledge levels actually increase after watching a video (ie, to study the efficacy) and which subgroups benefit most from Watchyourmeds. Eventually, another interesting direction for future research would be to examine the effectiveness of Watchyourmeds on clinical outcomes such as medication adherence and correct medication use.

### Conclusions

This study suggests that a web-based library with animated videos is a valuable and acceptable addition to stand-alone package leaflets to increase understanding and accessibility of medication information. However, the current research method only used self-reported data of users who watched at least 1 entire video on the Watchyourmeds platform. Further research is needed to establish the best practice for different types of users and to better understand the user perspectives, as well as the actual impact on users’ medication knowledge and clinical outcomes such as medication adherence and correct medication usage.

## Appendix

Multimedia Appendix 1 Screenshot of an example of an animated video.

Multimedia Appendix 2 Number of pharmacies participating, distributed videos and distribution methods.

Multimedia Appendix 3 Subgroup analyses based on gender, age and educational level.
