# Supplementary figures and images for: Using Animated Videos to Promote the Accessibility and Understandability of Package Leaflets: Retrospective Observational Study Evaluating the First Year of Implementation
Source: J Med Internet Res. 2023 May 4;25:e40914. doi: 10.2196/40914 (PMC10196893; doi:10.2196/40914)

**Multimedia Appendix 1.** Screenshot of an example of an animated video


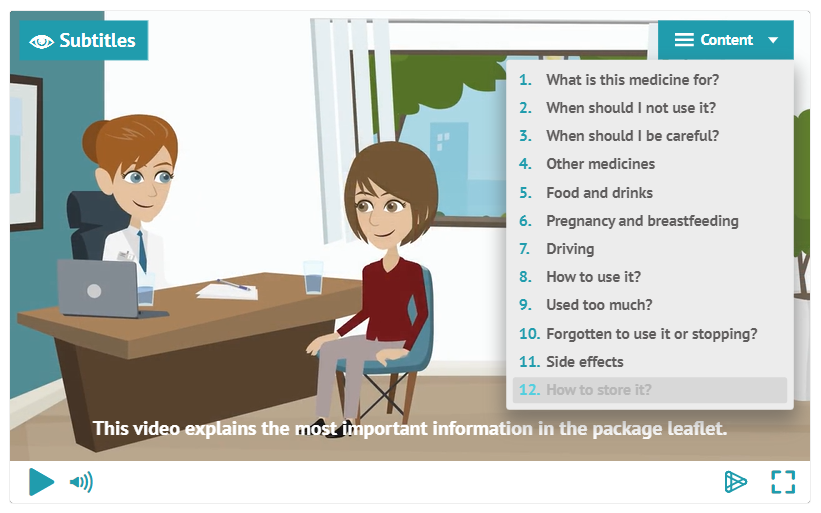

Supplement: Multimedia Appendix 1 [file jmir_v25i1e40914_app1.docx]
