# Supplementary material for: Using Animated Videos to Promote the Accessibility and Understandability of Package Leaflets: Retrospective Observational Study Evaluating the First Year of Implementation
Source: J Med Internet Res. 2023 May 4;25:e40914. doi: 10.2196/40914 (PMC10196893; doi:10.2196/40914)
Supplement: Multimedia Appendix 2 [file jmir_v25i1e40914_app2.docx]

**Multimedia Appendix 2 –** Figures number of pharmacies participating, distributed videos and distribution methods


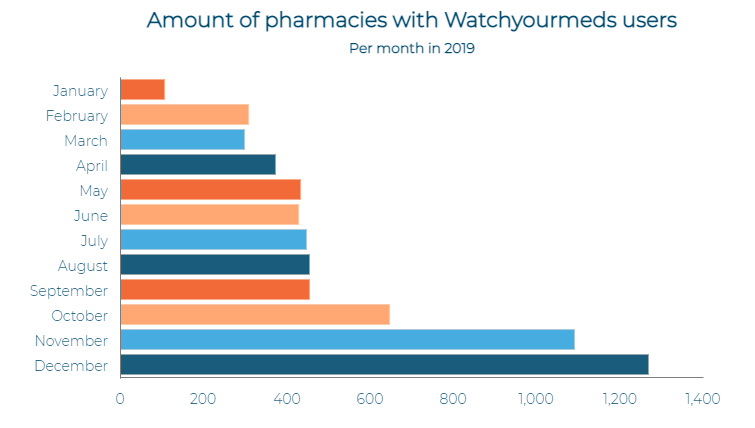


**Figure S1.** Amount of pharmacies with Watchyourmeds users


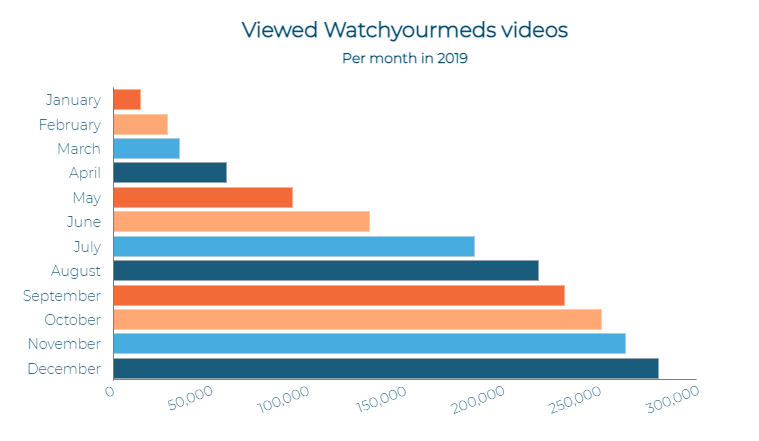


**Figure S2.** Viewed Watchyourmeds videos


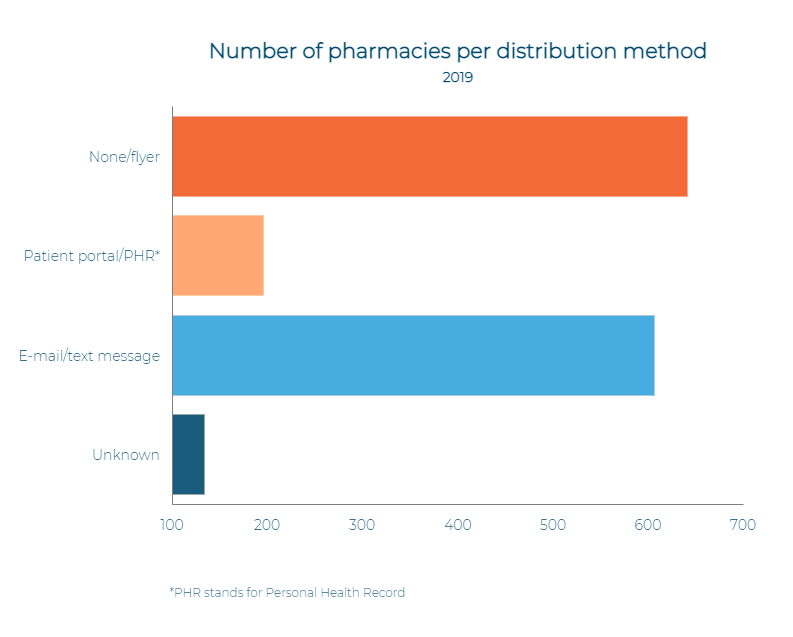


**Figure S3.** Number of pharmacies per distribution method
