# Supplementary material for: Using Animated Videos to Promote the Accessibility and Understandability of Package Leaflets: Retrospective Observational Study Evaluating the First Year of Implementation
Source: J Med Internet Res. 2023 May 4;25:e40914. doi: 10.2196/40914 (PMC10196893; doi:10.2196/40914)
Supplement: Multimedia Appendix 3 [file jmir_v25i1e40914_app3.docx]

**Multimedia Appendix 3** – Subgroup analyses based on gender, age and educational level

**Table S1.** Subgroup analyses (chi-squared tests) based on gender regarding the evaluation of online platform Watchyourmeds

| Question and answer options | | Female  N (%) | Male  N (%) |
| --- | --- | --- | --- |
| **To what extent did you understand the information in this**  **Watchyourmeds?** | | | |
|  | I did not understand any of it | 15a (0.6) | 26a (1.1) |
|  | I understood very little | 4a (0.2) | 8a (0.3) |
|  | I understood about half of it | 8a (0.3) | 8a (0.3) |
|  | I understood most of the information | 125a (5.2) | 167b (7.0) |
|  | I understood the information fully | 2257a (93.7) | 2181b (91.3) |
| **Did Watchyourmeds add to the information about your medication**  **that you received from your healthcare professional?** | | | |
|  | No | 732a (30.4) | 638b (26.7) |
|  | Yes | 1677a (69.6) | 1752b (73.3) |
| **Did you think there was information missing from this**  **Watchyourmeds?** | | |  |
|  | I did not think anything was missing | 1859a (81.8) | 1799a (80.3) |
|  | I felt very little was missing | 294a (12.9) | 338b (15.1) |
|  | I thought a number of things were missing | 104a (4.6) | 71b (3.2) |
|  | I missed a lot | 15a (0.7) | 31b (1.4) |
| **Are you going to take or use the medication for which you looked**  **at this Watchyourmeds?** | | | |
|  | I do not know | 106a (4.4) | 87a (3.6) |
|  | No | 44a (1.8) | 36a (1.5) |
|  | Yes | 2259a (93.8) | 2267a (94.9) |
| **After watching Watchyourmeds, do you think you have enough**  **knowledge to use this medicine properly?** | | | |
|  | I certainly know enough | 1899a (80.1) | 1845a (78.5) |
|  | I probably know enough | 437a (18.4) | 467a (19.9) |
|  | I still have too little knowledge | 35a (1.5) | 37a (1.6) |
| **Do you think you will use Watchyourmeds again, and also for other**  **medication?** | | | |
|  | No, I will never use Watchyourmeds again | 83a (3.4) | 72a (2.9) |
|  | I do not think I will use Watchyourmeds very often | 367a (14.8) | 262b (10.7) |
|  | Yes, I think I will usually use Watchyourmeds | 908a (36.7) | 875a (35.8) |
|  | Yes, I will use Watchyourmeds for all my medication | 1116a (45.1) | 1237b (50.6) |

Each subscript letter denotes a subset of the categories whose column proportions do not differ significantly from each other at the ,05 level.

**Table S2.** Subgroup analyses (chi-squared tests) based on age regarding the evaluation of online platform Watchyourmeds

| Question and answer options | | Old (≥ 55 years)  N (%) | Young (≤ 54 years)  N (%) |
| --- | --- | --- | --- |
| **To what extent did you understand the information in this**  **Watchyourmeds?** | | | |
|  | I did not understand any of it | 32a (0.9) | 6a (0.6) |
|  | I understood very little | 9a (0.3) | 3a (0.3) |
|  | I understood about half of it | 12a (0.3) | 2a (0.3) |
|  | I understood most of the information | 205a (5.9) | 66a (6.9) |
|  | I understood the information fully | 3200a (92.5) | 878a (91.9) |
| **Did Watchyourmeds add to the information about your medication**  **that you received from your healthcare professional?** | | | |
|  | No | 1036a (30.0) | 228b (23.9) |
|  | Yes | 2422a (70.0) | 727b (76.1) |
| **Did you think there was information missing from this**  **Watchyourmeds?** | | | |
|  | I did not think anything was missing | 2705a (83.2) | 663b (74.1) |
|  | I felt very little was missing | 409a (12.6) | 169b (18.9) |
|  | I thought a number of things were missing | 102a (3.1) | 53b (5.9) |
|  | I missed a lot | 34a (1.0) | 10a (1.1) |
| **Are you going to take or use the medication for which you looked**  **at this Watchyourmeds?** | | | |
|  | I do not know | 130a (3.8) | 50b (5.2) |
|  | No | 55a (1.6) | 22a (2.3) |
|  | Yes | 3273a (94.7) | 883b (92.5) |
| **After watching Watchyourmeds, do you think you have enough**  **knowledge to use this medicine properly?** | | | |
|  | I certainly know enough | 2736a (80.6) | 723b (76.3) |
|  | I probably know enough | 615a (18.1) | 199b (21.0) |
|  | I still have too little knowledge | 43a (1.3) | 26b (2.7) |
| **Do you think you will use Watchyourmeds again, and also for other**  **medication?** | | | |
|  | No, I will never use Watchyourmeds again | 114a (3.2) | 29a (3.0) |
|  | I do not think I will use Watchyourmeds very often | 423a (11.9) | 152b (15.6) |
|  | Yes, I think I will usually use Watchyourmeds | 1219a (34.3) | 405b (41.5) |
|  | Yes, I will use Watchyourmeds for all my medication | 1803a (50.7) | 389b (39.9) |

Each subscript letter denotes a subset of the categories whose column proportions do not differ significantly from each other at the ,05 level.

**Table S3.** Subgroup analyses (chi-squared tests) based on educational level regarding the evaluation of online platform Watchyourmeds

| Question and answer options | | No or elementary education  N (%) | Lower education  N (%) | Middle education  N (%) | Higher education  N (%) |
| --- | --- | --- | --- | --- | --- |
| **To what extent did you understand the**  **information in this Watchyourmeds?**^*^ | | | | | |
|  | I did not understand any of it | 4a (4.3) | 5b (0.4) | 9b (0.7) | 11b (0.8) |
|  | I understood very little | 0a (0.0) | 4a (0.3) | 1a (0.1) | 5a (0.4) |
|  | I understood about half of it | 2a (2.2) | 4b (0.3) | 4b (0.3) | 2b (0.2) |
|  | I understood most of the information | 13a (14.1) | 88b (6.5) | 77b,c (5.9) | 61c (4.6) |
|  | I understood the information fully | 73a (79.3) | 1254b (92.5) | 1209b (93.0) | 1235b (94.0) |
| **Did Watchyourmeds add to the information**  **about your medication that you received**  **from your healthcare professional?** | | | | | |
|  | No | 10a (10.9) | 289b (21.3) | 356c (27.4) | 516d (39.3) |
|  | Yes | 82a (89.1) | 1066b (78.7) | 944c (72.6) | 798d (60.7) |
| **Did you think there was information missing**  **from this Watchyourmeds?** | | | | | |
|  | I did not think anything was missing | 69a,b (82.1) | 1104b (85.6) | 984a (80.0) | 964a (78.4) |
|  | I felt very little was missing | 11a (13.1) | 161a (12.5) | 186a (15.1) | 171a (13.9) |
|  | I thought a number of things were missing | 2a,b,c (2.4) | 19c (1.5) | 52b (4.2) | 74a (6.0) |
|  | I missed a lot | 2a,b (2.4) | 6c (0.5) | 8b,c (0.7) | 20a (1.6) |
| **Are you going to take or use the medication**  **for which you looked at this Watchyourmeds?** | | | | | |
|  | I do not know | 4a,b (4.3) | 38b (2.8) | 49a,b (3.8) | 65a (4.9) |
|  | No | 3a,b (3.3) | 16a,b (1.2) | 15b (1.2) | 28a (2.1) |
|  | Yes | 85a,b (92.4) | 1301b (96.0) | 1236b (95.1) | 1221a (92.9) |
| **After watching Watchyourmeds, do you**  **think you have enough knowledge to use this**  **medicine properly?** | | | | | |
|  | I certainly know enough | 70a (78.7) | 1057a (79.1) | 1016a (78.8) | 1039a (80.9) |
|  | I probably know enough | 17a,b (19.1) | 269b (20.1) | 258b (20.0) | 218a (17.0) |
|  | I still have too little knowledge | 2a,b (2.2) | 10b (0.7) | 16a,b (1.2) | 28a (2.2) |
| **Do you think you will use Watchyourmeds**  **again, and also for other medication?** | | | | | |
|  | No, I will never use Watchyourmeds again | 7a (7.6) | 25b (1.8) | 33b (2.5) | 63a (4.8) |
|  | I do not think I will use Watchyourmeds very often | 2a (2.2) | 119b (8.8) | 147c (11.3) | 259d (19.7) |
|  | Yes, I think I will usually use Watchyourmeds | 19a (20.7) | 477b (35.2) | 525c (40.4) | 505b,c (38.4) |
|  | Yes, I will use Watchyourmeds for all my medication | 64a (69.6) | 734b (54.2) | 595c (45.8) | 487d (37.1) |

Each subscript letter denotes a subset of the categories whose column proportions do not differ significantly from each other at the ,05 level.

^*^ 9 cells (45.0%) have expected count less than 5.
